# Supplementary material for: GAF-CaMP3–sfGFP, An Enhanced Version of the Near-Infrared Genetically Encoded Positive Phytochrome-Based Calcium Indicator for the Visualization of Neuronal Activity
Source: Int J Mol Sci. 2020 Sep 19;21(18):6883. doi: 10.3390/ijms21186883 (PMC7555670; doi:10.3390/ijms21186883)
Supplement: Supplementary file 1 [file ijms-21-06883-s001.pdf]

Supplementary Information

GAF-CaMP3–sfGFP, An Enhanced Version of the Near-Infrared Genetically Encoded Positive Phytochrome-Based Calcium Indicator for the Visualization of Neuronal Activity

Oksana M. Subach, and Fedor V. Subach

Supplementary Tables and Figures

**Table S1**	**2**
**Figure S1**	**3**
**Figure S2**	**4**
**Figure S3**	**5**

Table S1. List of primers.

Primer	Primer sequence (5′-3′)
**GAF-BglII**	actagatctATGCGGGCAGGTCCATCC
**GAF-EcoRI-r**	gtagaattcAGACTCAAGGGCAGCTATTC
**mCherry-HindIII-r**	GATAAGCTTTTACTTATACAGCTCGTC
**GAF-EcoRI-r2**	GTAGAATTCTCAAGACTCAAGGGCAGCTATTC

NES-GAF-CaMP3-sfGFP-stop gene:

ATGCTTCAACTTCCTCCTCTTGAACGTCTTACTCTTTCGAGATCTATGCGGGCAGGTCCATCCATTG
ATCAGTCTGGAATGTTGACGCCCGCCCTTGAGAGGATTAGGGCTGCCGAGAGCCTTCGGGCCCTC
TGTGACGACACCGCTTTGCTTTTTCGACGATGTACCGGCTACGACCGTGTAATGGTGTACAGATTT
GGCGCTCATGGACAGGACCAGGTATTTAGTGAGTGTCGTGCGCCGGGACTGGAGAGCTACCTGG
GTAACCGCTACCCTAGCTCAATGGTTCCACGATTGGCAAGGCAACTGTATCTGCGGCAACGAGTT
CGAATGAGGGCGGACGTGGCTTACCTAGGGCGGCCAACGAGCCCTTTGCCGGACCAACTGACCG
AAGAGCAGATCGCAGAATTTAAAGAGGCTTTCACCCTATTTGACAAGGACGGGGATGGGACAGT
AACCACCAAGGAGCTGGGGGCGGTGATGCGGTCTCTGGGGCAGAACCCCACAGAAGCAGAGCT
GCGGGTCATGATCGATGAAGTAGATGCCGACGGTGACGGCACTCTCGACTGCCCTGAGTTCCTGG
CAATGATGGCAAGAAGAATGAAATACAGGGACACGGAAGAAGAAATTAGAGAAGCGTTCGGTG
TGGTTGATGCGGATGGCAATGGCTACATCAGTGCAGCAGAGCTTCGCCATGTGTTGACAAGCCTT
GGAGAGAAGTTAACAGATGAAGAGGTTGGTGAATTGATCAGGGAAGCAGCCATCAATGGGGAT
GGCCAGGTGAACTGCGAGGAGTTCGTACAAATGATGACAGCGAGGGGCGATTCAGGAGGCGGG
TGCTCATCGCGTCGTATGTGGAATAAGGTAGGTCACGCAGTCAGAGCTATAGGTCGGCTGAGCTC
CGGTGGTTGTCATCTCCGGTACCTCAATGATATGGGGGTCCGCGCCTCATTGGTTGTAAGCCTGGT
TGTAGGCGGAAAACTTTGGGGGCTGGTGGTGTGTTACCATTATCTTCCGAGATACATCCACCATG
AACTCAGGGCATATTGCAGAAGTCTTGCGGATGGAATTGCGGCGAGAATAGCTGCCCTTGAGTCT
GAATTCATGGTGAGCAAGGGCGAGGAGCTGTTCACCGGGGTGGTGCCCATCCTGGTCGAGCTGG
ACGGCGACGTAAACGGCCACAAGTTCAGCGTGCGCGGCGAGGGCGAGGGCGATGCCACCAACG
GCAAGCTGACCCTGAAGTTCATCTGCACCACCGGCAAGCTGCCCGTGCCCTGGCCCACCCTCGTG
ACCACCCTGACCTACGGCGTGCAGTGCTTCAGCCGCTACCCCGACCACATGAAGCGCCACGACT
TCTTCAAGTCCGCCATGCCCGAAGGCTACGTCCAGGAGCGCACCATCAGCTTCAAGGACGACGG
CACCTACAAGACCCGCGCCGAGGTGAAGTTCGAGGGCGACACCCTGGTGAACCGCATCGAGCTG
AAGGGCATCGACTTCAAGGAGGACGGCAACATCCTGGGGCACAAGCTGGAGTACAACTTCAAC
AGCCACAACGTCTATATCACCGCCGACAAGCAGAAGAACGGCATCAAGGCCAACTTCAAGATC
CGCCACAACGTGGAGGACGGCAGCGTGCAGCTCGCCGACCACTACCAGCAGAACACCCCCATC
GGCGACGGCCCCGTGCTGCTGCCCGACAACCACTACCTGAGCACCCAGTCCGTGCTGAGCAAAG
ACCCCAACGAGAAGCGCGATCACATGGTCCTGCTGGAGTTCGTGACCGCCGCCGGGATCACTCA
CGGCATGGACGAGCTGTATAAGTAA

Figure S1. Nucleotide sequence of NES-GAF-CaMP3-sfGFP protein.

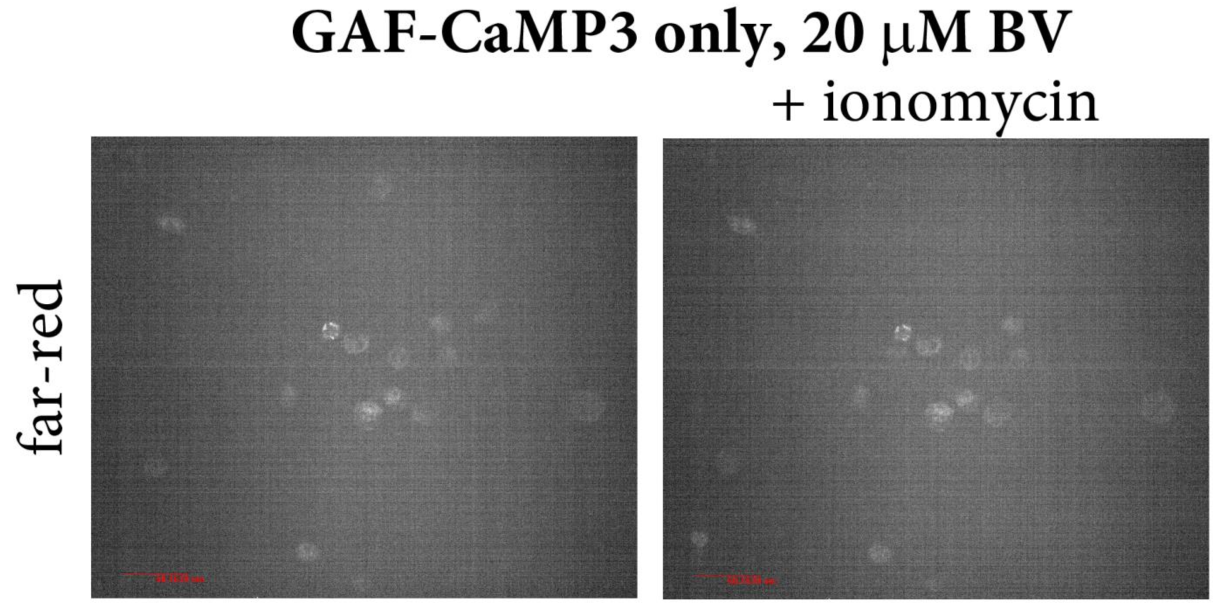

Figure S2. Expression of the GAF-CaMP3 indicator and its response to Ca^{2+} variations in HeLa cells. Confocal images of HeLa cells expressing the NES-GAF-CaMP3 calcium indicator (with deletion of sfGFP on its C-terminal end) in the presence of 20 μM external BV before and after addition of 2.5 μM ionomycin. Far-red fluorescence channel correspond to Ex640nm/Em685/40nm. Scale bar, 50 μm.

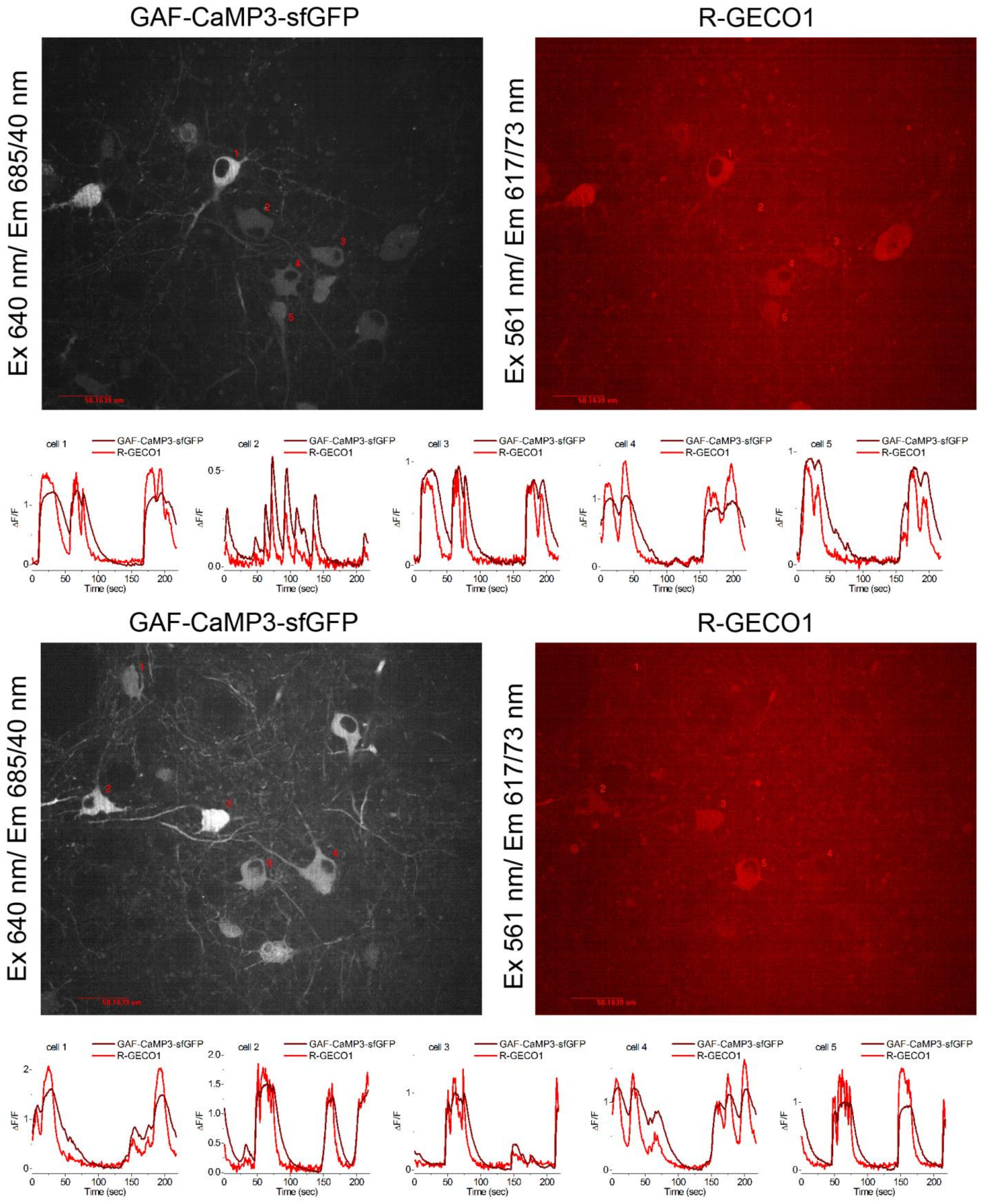

Figure S3. Calcium imaging of non-specific (spontaneous) activity of neuronal cultures co-expressing the GAF-CaMP3-sfGFP and R-GECO1 calcium indicators. Confocal images of two regions of interest for neuronal cultures co-expressing GAF-CaMP3-sfGFP and R-GECO1 indicators in the presence of 10 μM external BV. Scale bar, 50 μm. Examples of ΔF/F traces for the 5 cells are shown for each of two regions of interest. 10 μM BV was supplied 5-24 hours before imaging. Neuronal cultures co-expressing the NES-GAF-CaMP3-sfGFP and NES-R-GECO1 indicators were imaged on DIV 15th. Neuronal cultures were transduced on DIV 4th with the mixture of rAAVs carrying NES-GAF-CaMP3-sfGFP and NES-R-GECO1.
